# Case Report: pulmonary MALT lymphoma with *BIRC3::MALT1* fusion and indeterminate PET/CT findings

**DOI:** 10.3389/fonc.2026.1887841

**Published:** 2026-07-17

**Authors:** Hongliang Zhang, Helun Cai

**Affiliations:** Department of Geriatrics, Xiangyang No.1 People’s Hospital, Hubei University of Medicine, Xiangyang, China

**Keywords:** *API2-MALT1*, *BIRC3::MALT1*, case report, extranodal marginal zone lymphoma, PET/CT, pulmonary MALT lymphoma, R-CVP

## Abstract

**Background:**

Pulmonary extranodal marginal zone lymphoma of mucosa-associated lymphoid tissue (pulmonary MALT lymphoma) is an indolent B-cell lymphoma that can mimic infection, inflammatory disease, or epithelial malignancy. Indeterminate fluorodeoxyglucose-avid abnormalities may complicate staging and lead to overinterpretation of disease extent.

**Case presentation:**

A 49-year-old man with no smoking history underwent video-assisted thoracoscopic right lower-lobe superior segmentectomy for a 12 × 9 mm pulmonary nodule. Histopathology and immunohistochemistry supported pulmonary MALT lymphoma, and fluorescence *in situ* hybridization detected a *BIRC3::MALT1* (formerly API2-*MALT1*) fusion in 35% of analyzed cells. Resection margins were negative; the only separately documented LN7 lymph node showed reactive hyperplasia without lymphoma, and bone marrow examination was negative. PET/CT performed 11 days after surgery showed expected postoperative inflammatory uptake, mildly avid right upper-lobe ground-glass opacities (SUVmax 1.0), and circumferential thickening of the horizontal duodenum (SUVmax 4.0). Review of serial preoperative CT reports showed that the additional right-lung ground-glass opacities had been present since at least January 2021 and were largely stable through March 2025. Gastroscopy showed no duodenal mucosal abnormality, no biopsy was obtained, and the duodenal finding remained essentially unchanged after rituximab-based systemic therapy. The minimum confirmed extent was therefore a resected pulmonary MALT lymphoma without proven nodal, marrow, additional pulmonary, or gastrointestinal involvement.

**Conclusion:**

Mild FDG uptake in longstanding pulmonary opacities and an unbiopsied gastrointestinal abnormality should not be interpreted as lymphoma dissemination. Histopathology, immunophenotyping, molecular testing, longitudinal imaging, and pathologic confirmation of suspected additional sites should be integrated before disease extent and treatment response are assigned.

## Introduction

Extranodal marginal zone lymphoma of mucosa-associated lymphoid tissue (MALT lymphoma) is an indolent B-cell non-Hodgkin lymphoma that most often arises in the stomach but may involve the lung and other extranodal organs (1–3). Pulmonary MALT lymphoma is the most common primary pulmonary lymphoma, although it remains uncommon overall. Patients may be asymptomatic or have nonspecific respiratory symptoms, and CT findings include solitary or multiple nodules, consolidation, air bronchograms, and ground-glass opacities (4, 5). Because these appearances overlap with infection, inflammatory disease, and epithelial malignancy, diagnosis and staging require tissue sampling and integration of morphology, immunophenotype, molecular findings, and longitudinal imaging.

The *BIRC3::MALT1* fusion, historically termed API2-*MALT1* and generated by t(11;18)(q21;q21), is a recognized molecular abnormality in MALT lymphoma and activates nuclear factor-κB signaling (6–8). Its detection can support an integrated diagnosis but is not a stand-alone indication for systemic treatment. We report a pulmonary MALT lymphoma with *BIRC3::MALT1* fusion in which postoperative PET/CT initially raised concern for additional pulmonary and duodenal disease. Retrospective review of the longitudinal CT record and subsequent follow-up demonstrate why these abnormalities should remain indeterminate rather than be labeled multifocal or disseminated lymphoma.

## Case description

A 49-year-old man was admitted to thoracic surgery on March 17, 2025, after surveillance imaging showed a right lower-lobe pulmonary nodule. He reported mild cough with sputum but denied fever, chills, abdominal pain, diarrhea, appetite change, or weight loss. He had no smoking or alcohol-drinking history, no known autoimmune disease, and no family history of lymphoma, lung cancer, or other malignant tumors. His history included occupational welding exposure, hypertension, prior hepatitis B virus (HBV) infection, previous antituberculosis treatment after detection of a pulmonary nodule, and resection of a left thyroid microcarcinoma followed by levothyroxine therapy. The complete clinical timeline is summarized in **Table 1**.

**Table 1:** Timeline of clinical presentation, diagnostic assessment, treatment, and follow-up.

Time point	Clinical information
Jan 2021-Mar 2025	Serial chest CT reports documented longstanding right-lung ground-glass opacities that were largely stable.
Mar 17, 2025	Thoracic surgery admission for a right lower-lobe 12 × 9 mm pulmonary nodule; mild cough and sputum, without fever or weight loss.
Mar 20, 2025	Video-assisted thoracoscopic right lower-lobe superior segmentectomy with hilar/mediastinal lymph-node sampling; frozen section favored a lymphoid proliferative lesion.
Mar 24, 2025	Final pathology: pulmonary low-grade B-cell lymphoma consistent with MALT lymphoma; margins and pleura uninvolved; LN7 showed reactive hyperplasia with anthracotic pigment deposition and no lymphoma (0/1).
Mar 31, 2025	PET/CT 11 days after surgery showed postoperative inflammatory uptake, minimally avid right upper-lobe ground-glass opacities, and an FDG-avid duodenal abnormality.
Apr 2, 2025	Gastroscopy showed erosive gastritis and no obvious duodenal mucosal abnormality; duodenal biopsy was not obtained.
Apr 15 and May 6, 2025	Cycles 1–2 R-CVP.
May 28-29, 2025	Abdominal MRI described persistent/apparently increased duodenal abnormality; treatment was changed to R-CHOP.
Jun 19, 2025	Cycle 4 R-CHOP.
After 4 cycles	The treating team recorded stable disease; orelabrutinib 150 mg orally once daily was started.
Sep 15, 2025	PET/CT showed no abnormally increased pulmonary FDG uptake and less conspicuous right-lung opacities; their relationship to lymphoma remained unproven. The duodenal abnormality persisted and was essentially unchanged.
Dec 8, 2025	Gastroscopy again documented no biopsy-proven duodenal lymphoma.
May 1, 2026	At approximately 1 year, the patient was clinically asymptomatic; no clinical progression of the pathologically confirmed pulmonary lymphoma was identified, while the duodenal abnormality remained unconfirmed.

Physical examination showed no palpable superficial lymphadenopathy or hepatosplenomegaly. Breath sounds were clear, and the abdomen was soft and non-tender. Pulmonary function testing demonstrated moderate obstructive ventilatory impairment: forced expiratory volume in 1 s (FEV1), 1.88 L; FEV1 percentage predicted, 65.3%; forced vital capacity, 2.81 L; FEV1/FVC, 66.95%; and maximal voluntary ventilation percentage predicted, 89.7%.

A preoperative chest CT on March 17, 2025, showed a right lower-lobe part-solid/high-density nodule measuring 12 × 9 mm with indistinct margins and no abnormal enhancement. Additional right-lung ground-glass opacities and a stable left upper-lobe solid nodule were also reported. A retrospective review of the available serial CT reports showed that right-lung ground-glass opacities had been present since at least January 2021 and were repeatedly described as unchanged or essentially unchanged through May 2024. In January 2025, two right-lung ground-glass lesions were described as slightly fuller than on the preceding examination, whereas the March 2025 preoperative report again characterized them as essentially unchanged and without abnormal enhancement. Thus, the additional opacities predated surgery by several years and did not represent newly appearing postoperative lesions. The baseline CT appearance of the resected right lower-lobe nodule is shown in **Figure 1**.

**Figure 1:**
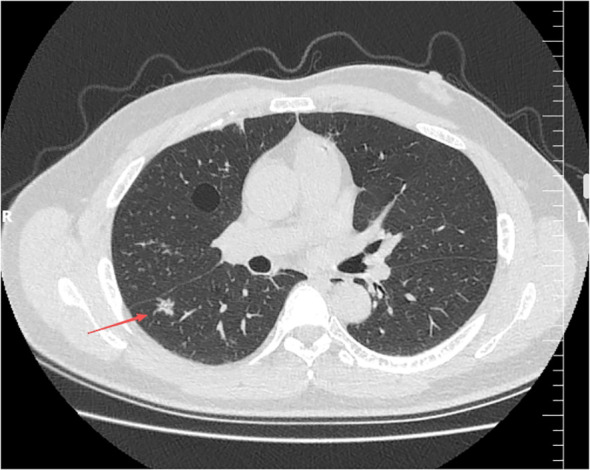
Initial imaging findings. Baseline chest CT showed a right lower-lobe part-solid/high-density nodule measuring 12 mm × 9 mm with indistinct borders.

## Pathological and molecular assessment

On March 20, 2025, the patient underwent video-assisted thoracoscopic right lower-lobe superior segmentectomy with hilar and mediastinal lymph-node sampling. Intraoperative frozen section suggested a lymphoid proliferative lesion. Gross examination showed a 1.0 × 1.8 × 0.7 cm gray-white lesion in the resected segment. The lesion was approximately 2.8 cm from the stapled margin, 3.5 cm from the bronchial margin, and 0.2 cm from the visceral pleura. The bronchial stump, visceral pleura, and stapled margin were uninvolved. The only separately documented nodal specimen, LN7, showed reactive hyperplasia with anthracotic pigment deposition and no lymphoma (0/1).

Histopathology and immunohistochemistry supported extranodal marginal zone lymphoma of mucosa-associated lymphoid tissue. Tumor cells were positive for CD20, CD79a, CD43, BCL2, MNDA, and BCL10; BCL6 showed scattered positivity. The cells were negative for CD3, CD5, CD10, CD23, cyclin D1, SOX11, and IgD. Follicular dendritic-cell meshworks were highlighted by CD21 and CD23. The Ki-67 proliferation index was approximately 5%, and Epstein-Barr virus-encoded RNA *in situ* hybridization was negative. Serum protein electrophoresis and immunofixation did not identify a monoclonal protein. Representative histopathological and immunohistochemical findings are shown in **Figure 2**.

**Figure 2:**
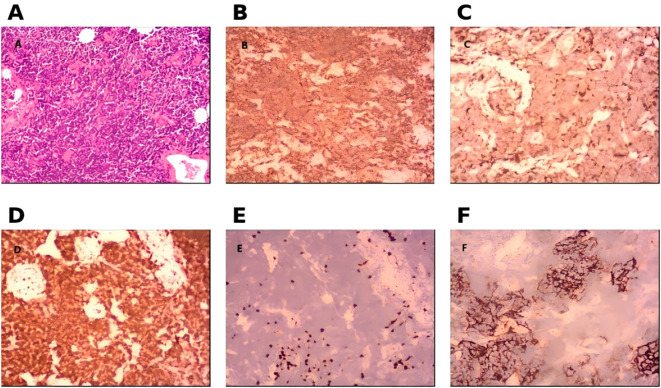
Histopathology and immunohistochemistry. **(A)** Hematoxylin and eosin staining. **(B)** CD20. **(C)** CD43. **(D)** BCL2. **(E)** Ki-67. **(F)** Follicular dendritic-cell meshwork highlighted by CD21. IHC, immunohistochemistry.

Fluorescence *in situ* hybridization was performed on formalin-fixed paraffin-embedded tissue using an API2/*MALT1* dual-color fusion probe (Anbiping). API2/*BIRC3* at 11q22 was labeled red and *MALT1* at 18q21 was labeled green. Hybridization was performed with a ThermoBrite system; signals were evaluated by fluorescence microscopy with dual-person technical review and appropriate positive and negative controls. The abnormal 2F1R1G signal pattern was present in 35.0% of analyzed cells, exceeding the laboratory positivity threshold of 5.0%, and was interpreted as *BIRC3::MALT1* fusion positive. The original pathology report did not explicitly document lymphoepithelial lesions, bronchus-associated lymphoid tissue distribution, plasmacytic differentiation, light-chain restriction, or immunoglobulin heavy-chain gene rearrangement testing.

## Diagnostic assessment and staging

Laboratory tests obtained on postoperative day 1 (March 21, 2025) showed lactate dehydrogenase (LDH) 408 U/L, fibrinogen 4.91 g/L, D-dimer 1.07 mg/L, and antithrombin III 77.3%. Because these measurements were obtained immediately after thoracic surgery, the LDH and coagulation abnormalities were considered potentially confounded by tissue injury, inflammation, or hemolysis and were not used as evidence of aggressive or disseminated lymphoma. Before anti-CD20 therapy, HBV testing showed HBsAg <0.05 IU/mL, anti-HBs >1000 mIU/mL, HBeAg <0.10 S/CO, anti-HBe 0.14 S/CO, anti-HBc 21.68 S/CO, normal transaminases, and HBV DNA below the detection threshold; prophylactic entecavir was administered.

PET/CT on March 31, 2025, 11 days after surgery, showed metabolically active changes in the operative bed and right chest wall that were interpreted as postoperative inflammation. Multiple right upper-lobe ground-glass opacities had only mild FDG uptake; the largest was approximately 0.7 cm with SUVmax 1.0. In view of their documented presence for several years and overall stability on serial preoperative CT reports, these lesions were classified as longstanding indeterminate pulmonary opacities rather than evidence of multifocal pulmonary MALT lymphoma.

PET/CT also showed circumferential thickening of the horizontal duodenum with SUVmax 4.0. Gastroscopy on April 2, 2025, showed erosive gastritis but no obvious duodenal mucosal abnormality, and neither duodenal biopsy nor endoscopic ultrasound-guided sampling was performed. Bone marrow cytology and biopsy showed no lymphoma involvement. The minimum confirmed disease extent was therefore a completely resected pulmonary MALT lymphoma without proven nodal or marrow involvement. Under the Ann Arbor/Lugano framework, the pathologically confirmed disease was assigned a working stage IE, while recognizing that nodal assessment was limited to the documented LN7 specimen and that the additional pulmonary and duodenal findings were not pathologically characterized.

## Therapeutic intervention

The patient recovered from surgery but reported fatigue and night sweats in mid-April 2025, without documented fever or weight loss. At that time, the treating team selected systemic therapy because of the new symptoms, limited pulmonary reserve, and concern that the indeterminate pulmonary and duodenal abnormalities might represent additional disease. This decision was made in the setting of diagnostic uncertainty. Neither mild pulmonary FDG uptake, the duodenal PET/CT finding, postoperative LDH elevation, nor *BIRC3::MALT1* positivity independently established dissemination or constituted a standard indication for chemotherapy.

The patient received two cycles of R-CVP on April 15 and May 6, 2025: rituximab 630 mg on day 0, cyclophosphamide 1,200 mg on day 1, vincristine 2 mg on day 1, and prednisone 100 mg on days 1-5. After abdominal magnetic resonance imaging on May 28 described persistent and apparently more extensive duodenal wall abnormality, the regimen was changed at the treating team’s discretion to R-CHOP on May 29 and June 19, 2025: rituximab 640 mg on day 0, cyclophosphamide 1,200 mg on day 1, doxorubicin 70 mg on day 1, vincristine 2 mg on day 1, and prednisone 100 mg on days 1-5. After four cycles, the treating team recorded the overall radiologic assessment as stable disease, and orelabrutinib 150 mg orally once daily was subsequently started. Entecavir prophylaxis was continued. This treatment sequence is reported as the actual clinical course and should not be interpreted as a generalizable strategy for localized or *BIRC3::MALT1*-positive pulmonary MALT lymphoma.

## Follow-up and outcomes

Follow-up included chest CT and abdominal magnetic resonance imaging on May 26, 2025; chest CT on June 17; chest CT and abdominal magnetic resonance imaging on July 10; PET/CT on September 15; gastroscopy on December 8; and chest CT plus abdominal magnetic resonance imaging on May 1, 2026. The September PET/CT showed no abnormally increased pulmonary FDG uptake and near-complete resolution of the previously described right-lung opacities. Because those opacities had been longstanding, were not biopsied, and had shown only minimal baseline uptake, their subsequent radiologic change cannot retrospectively establish lymphoma involvement or be used as proof of lymphoma response.

In contrast, thickening and FDG uptake in the horizontal duodenum remained essentially unchanged from the March PET/CT. Repeat gastroscopy in December 2025 again did not document biopsy-proven duodenal lymphoma. The absence of a clear treatment-associated response further weakened the attribution of this lesion to MALT lymphoma. At approximately 1 year of follow-up, the patient denied fever, cough, sputum, chest discomfort, abdominal pain, abdominal distension, or diarrhea, and no clinical progression of the pathologically confirmed pulmonary lymphoma was identified. A comparison of the March 31 and September 15 PET/CT findings is shown in **Figure 3**.

**Figure 3:**
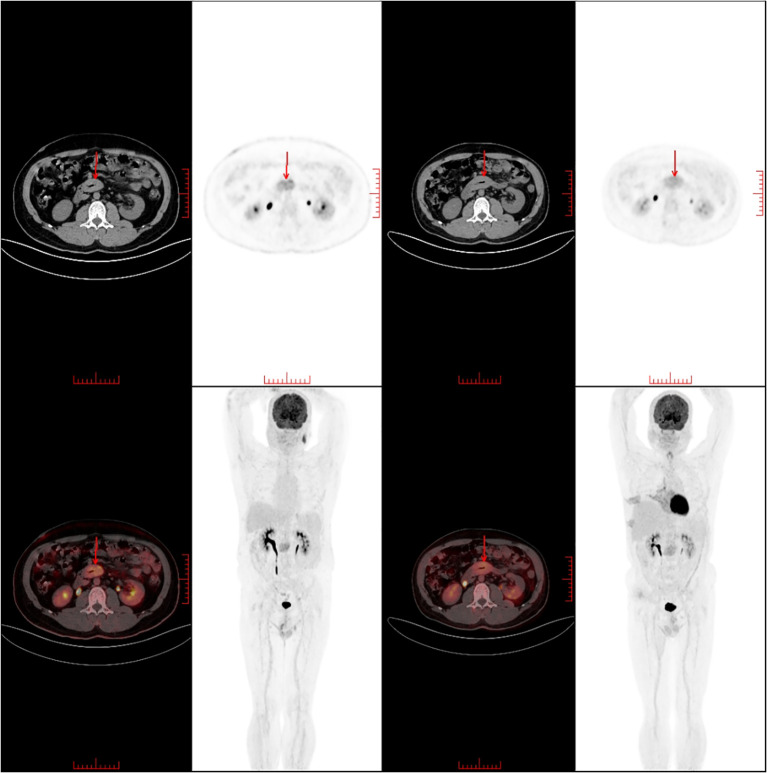
Follow-up imaging studies. PET/CT on September 15, 2025 (left) showed persistent horizontal duodenal wall thickening and FDG uptake that were essentially unchanged compared with PET/CT on March 31, 2025 (right).

## Discussion

This case has a focused diagnostic message: pulmonary MALT lymphoma requires tissue-based confirmation, whereas mildly FDG-avid abnormalities at other sites should not be incorporated into staging without compatible longitudinal behavior or pathologic evidence. The resected right lower-lobe lesion was supported by an indolent B-cell immunophenotype, exclusion of major small B-cell lymphoma mimics, a low Ki-67 index, and *BIRC3::MALT1* fusion. Molecular testing strengthened the integrated diagnosis but did not replace morphology or define the indication for treatment (6–8).

The serial preoperative CT record directly addresses the concern that the postoperative PET/CT represented multifocal pulmonary dissemination. Right-lung ground-glass opacities were documented from January 2021 and were repeatedly reported as stable over several years. Mild uptake with SUVmax 1.0 on PET/CT obtained only 11 days after surgery was therefore insufficient to reclassify these lesions as lymphoma. Pulmonary MALT lymphoma can present with ground-glass opacity, but this pattern is nonspecific, and both CT morphology and FDG uptake overlap with inflammatory and benign conditions (4, 9, 10). The appropriate conclusion is not that the opacities were definitively benign, but that lymphoma involvement was unproven.

The duodenal abnormality illustrates a similar problem. FDG uptake in the gastrointestinal tract may be physiologic or associated with inflammation, infection, and benign subepithelial processes (9–11). In this patient, conventional gastroscopy showed no corresponding mucosal lesion, no tissue sampling was obtained, and the imaging abnormality remained essentially unchanged after rituximab-containing therapy. These findings do not establish duodenal MALT lymphoma and do not support retrospective assignment of stage IV disease. Endoscopic ultrasound or image-guided tissue acquisition would have been more informative if clinically feasible.

Management of pulmonary MALT lymphoma should be individualized according to confirmed disease extent, symptoms, progression, organ compromise, and feasibility of observation or local therapy (12–15). The systemic treatment used in this case arose from contemporaneous clinical concern rather than proof of multifocal or gastrointestinal lymphoma. Reporting the sequence transparently is important, but its inclusion should not imply that *BIRC3::MALT1* positivity, transient postoperative LDH elevation, or indeterminate PET/CT findings are accepted indications for R-CVP, R-CHOP, or a Bruton tyrosine kinase inhibitor. The major clinical lesson is that uncertainty in staging should prompt targeted confirmation and surveillance rather than stronger diagnostic language.

This report has several limitations. Only one lymph-node specimen had a clearly documented pathologic result, the classical morphologic features of MALT lymphoma were incompletely detailed in the original pathology report, and neither the pulmonary ground-glass opacities nor the duodenal lesion was biopsied. These limitations require cautious staging and prevent attribution of the indeterminate abnormalities to lymphoma.

## Conclusion

The confirmed diagnosis was a resected pulmonary MALT lymphoma with *BIRC3::MALT1* fusion. Longstanding right-lung ground-glass opacities with minimal FDG uptake and a persistent unbiopsied duodenal abnormality did not provide sufficient evidence of multifocal or disseminated disease. Careful review of preoperative imaging, recognition of postoperative PET/CT confounding, and pathologic confirmation of suspected additional sites are essential to avoid over-staging and therapeutic overinterpretation.

## Data Availability

The original contributions presented in the study are included in the article/supplementary material. Further inquiries can be directed to the corresponding author.
